# Detection of genomic regions that differentiate *Bos indicus* from *Bos taurus* ancestral breeds for milk yield in Indian crossbred cows

**DOI:** 10.3389/fgene.2022.1082802

**Published:** 2023-01-09

**Authors:** Mohammad Al Kalaldeh, Marimuthu Swaminathan, Vinod Podtar, Santoshkumar Jadhav, Velu Dhanikachalam, Akshay Joshi, John P. Gibson

**Affiliations:** ^1^ Centre for Genetic Analysis and Applications, School of Environmental and Rural Science, University of New England, Armidale, NSW, Australia; ^2^ BAIF Development Research Foundation and Central Research Station, Pune, Maharashtra, India

**Keywords:** GWAS, milk yield, crossbred cows, breed origin, *Bos indicus*, *Bos taurus*

## Abstract

**Introduction:** In India, crossbred cows incorporate the high production of *B. taurus* dairy breeds and the environmental adaptation of local *B. indicus* cattle. Adaptation to different environments and selection in milk production have shaped the genetic differences between *B. indicus* and *B. taurus* cattle. The aim of this paper was to detect, for milk yield of crossbred cows, quantitative trait loci (QTL) that differentiate *B. indicus* from *B. taurus* ancestry, as well as QTL that are segregating within the ancestral breeds.

**Methods:** A total of 123,042 test-day milk records for 4,968 crossbred cows, genotyped with real and imputed 770 K SNP, were used. Breed origins were assigned to haplotypes of crossbred cows, and from that, were assigned to SNP alleles.

**Results:** At a false discovery rate (FDR) of 30%, a large number of genomic regions showed significant effects of *B. indicus* versus *B. taurus* origin on milk yield, with positive effects coming from both ancestors. No significant regions were detected for Holstein Friesian (HF) versus Jersey effects on milk yield. Additionally, no regions for SNP alleles segregating within indigenous, within HF, and within Jersey were detected. The most significant effects, at FDR 5%, were found in a region on BTA5 (43.98–49.44 Mbp) that differentiates *B. indicus* from *B. taurus*, with an estimated difference between homozygotes of approximately 10% of average yield, in favour of *B. indicus* origin.

**Discussion:** Our results indicate that evolutionary differences between *B. indicus* and *B. taurus* cattle for milk yield, as expressed in crossbred cows, occur at many causative loci across the genome. Although subject to the usual first estimation bias, some of the loci appear to have large effects that might make them useful for genomic selection in crossbreds, if confirmed in subsequent studies.

## 1 Introduction

India is the world’s largest producer of milk, producing more than 209 million tonnes per annum, with an annual growth of 5.8%. Just above half of the total liquid milk production is contributed by cattle, of which 55% are crossbreds between indigenous *Bos indicus* and exotic *Bos taurus* dairy breeds, with an average yield of 7.22 kg/day, and an average herd size of two cows (DAHD, 2021). Crossbred cows are used in India to combine the production potential of *B. taurus* dairy cattle with the adaptation to difficult environments of local *B. indicus* cattle. The estimated divergence time between *B. indicus* and *B. taurus* from a common ancestor varies from 200,000 to 575,000 years (Loftus et al., 1994a; Loftus et al., 1994b; Bradley et al., 1996), or even 2.0 million years ago (Hiendleder et al., 2008), based on mtDNA data, and from 610,000 to 850,000 years based on microsatellite data (MacHugh et al., 1997). Generally, both subspecies occupy distinct environmental and geographic locations worldwide (Troy et al., 2001; Orozco-terWengel et al., 2015). *B. taurus* cattle are usually found in temperate environments, whereas *B. indicus* cattle are highly adapted to tropical environments.

Given the time since the divergence, and the different environments in which they evolved, the two subspecies differ substantially for many adaptation traits such as heat tolerance and disease resistance (Barendse, 2017), and there is emerging evidence of different functional genetic variation between the two subspecies (Bolormaa et al., 2011; Bolormaa et al., 2013; Naval-Sánchez et al., 2020). It is likely that the subspecies are fixed or close to fixed for different alleles at many loci affecting traits that differentiate the subspecies. It is also likely that different quantitative trait loci (QTL) contribute to the genetic variation within *B. indicus versus* within *B. taurus* cattle. And, where the same QTL are segregating in the two subspecies, the phase and/or strength of linkage disequilibrium (LD) between QTL and SNP is not expected to be the same in both.

In crossbred cattle, SNP alleles can be assigned to their ancestral breed origin based on the inferred origin of the phased haplotypes in which they sit. This provides an opportunity to detect QTL that are segregating in crossbred cattle that are fixed for alternative alleles in the ancestral breeds and also detect QTL that are segregating within the ancestral breeds. The aim of this paper is to undertake a genome-wide association study (GWAS) for milk yield in crossbred cows that separates the effect of each SNP into: 1) the effect of indigenous *B. indicus versus* exotic *B. taurus* origin (breed origin effect) 2) the effect of QTL in LD with SNP alleles segregating within *B. indicus* ancestral breeds, and 3) the effect of QTL in LD with SNP alleles segregating within *B. taurus* ancestral breeds.

## 2 Materials and methods

### 2.1 Phenotypes

Phenotypes were collected within the Enhanced Genetic Gains program (EGP) of BAIF Development Research Foundation (baif.org.in) between 2016 and 2020. A total of 12,004 crossbred cows with four or more monthly test-day (TD) milk yield between 8 and 340 days after calving, in at least one lactation, were retained. Cows were crossbreds, of unknown breed compositions, between local indigenous *B. indicus* cattle and exotic *B. taurus* breeds. In the regions sampled, Holstein-Friesian (HF) and Jersey were the only *B. taurus* breeds known to have been used for crossbreeding. After data quality control (QC) (Al Kalaldeh et al., 2021), 11,510 crossbred cows with 223,379 TD yield in 5,305 herds remained. The number of crossbred cows per herd ranged from 1 to 44, with an average of 2.17. To obtain adjusted TD milk yield, TD records were corrected for fixed effects using a mixed linear model as described in (Al Kalaldeh et al., 2021). Fixed effects included parity, CDC (Cattle Development Centre), year-month (2016-09–2020-07), interaction of CDC and year-month, milk curve for each parity modelled by the third order Legendre polynomial (LP), milk curve for each CDC modelled by the third order LP. Random effects included the animal and herd effects under a repeatability model. The adjusted TD yield were obtained as the estimated animal effects plus the corresponding herd and residual effects.

### 2.2 Genotypes

The genotype data included 5,280 crossbred cows, of which 4,637 were genotyped with the GeneSeek Genomic Profiler (GGP) Bovine 50K BeadChip (Neogen GeneSeek Operations, Lincoln, NE, United States) and 643 genotyped with the Illumina 777k BovineHD BeadChip (Illumina Inc., San Diego, CA, United States). Additional genotype data were available for 198 pure HF, 175 pure Jersey, and 158 pure indigenous animals (bulls and cows) from the BAIF bull stud herd, 389 of which were genotyped with the Illumina BovineSNP50 chip and the remaining genotyped with the GGP Bovine 50K chip. SNP positions were assigned according to ARS-UDC1.2 assembly of the bovine reference genome (GCA_002263795.2). After being subject to QC as described by (2021), animals genotyped with the 50K chip were imputed to high density (HD) using reference samples that had real HD genotypes. The reference population for imputation included 684 pure Jerseys, and 968 pure Holsteins from the Bovine HapMap (Consortium et al., 2009), 666 pure Indian *B. indicus* indigenous animals from a separate BAIF project (Strucken et al., 2021), and the 643 crossbred cows from the BAIF EGP. The HapMap and the Indian indigenous samples were genotyped with the Illumina 777k assay and genotypes were received post-QC. Genotype imputation was carried out using the software FImpute (Sargolzaei et al., 2014), which resulted in 697,736 loci across the 29 *B. taurus* autosomes (BTA). After applying genotype QC and retaining cows with milk yield that passed the phenotype QC, 4,968 genotyped crossbred cows with 123,042 TD yield were available for GWAS.

The reference samples used to infer the local ancestry of crossbred cow haplotypes included indigenous, HF and Jersey ancestral breeds. The indigenous samples consisted of 95 animals selected from the pure Indian *B. indicus* indigenous samples, representing the least related animals within a larger sample, based on low genomic relationships (Aliloo et al., 2018). The number of animals selected from each breed was proportionate to the total number of animals available for each breed. The HF samples consisted of 21 Friesian animals from the Scottish Rural University College (SRUC), 21 Holstein animals from HapMap (Consortium et al., 2009), and 178 animals randomly selected from the purebred HF bulls in the BAIF bull stud. The Jersey samples included 21 Jersey animals from HapMap (Consortium et al., 2009) and 155 animals randomly selected from purebred Jerseys in the BAIF bull stud. The remaining HF and Jersey bull stud animals and all indigenous bull stud animals were used to validate the local ancestry assignments. All indigenous, HF, and Jersey bull stud animals have pedigree records and were confirmed by an ADMIXTURE analysis (Alexander et al., 2009) to have more than 98% purity. The HapMap and SRUC samples were genotyped using the Illumina 777k assay and data were received post-QC.

### 2.3 Assigning breed origin to alleles in crossbred cows

The assignment of breed origin to SNP alleles in crossbred cows was undertaken using three steps. First, the genotypes of reference animals and crossbred cows were phased using Eagle software (Loh et al., 2016) to provide haplotypes for local ancestry inference. Phasing was based on 676,577 SNPs common between both the reference animals and crossbred cows that had minor allele frequencies (MAF) higher than 1%.

Then, the breed origin of either two ancestors (*B. indicus* and *B. taurus*) or of three ancestors (*B. indicus*, HF, and Jersey) were assigned to the phased haplotypes using PCAdmix software (Brisbin et al., 2012). PCAdmix uses a window-based hidden Markov model (HMM) to trace the breed origin of haplotypes in the admixed populations. Different window sizes, ranging from 25 SNP to 1500 SNP, were used to determine the appropriate size to run PCAdmix. This was validated using the pure *B. indicus* and pure *B. taurus* (HF and Jersey) validation samples. The last window in the chromosome was excluded if it contained less than 80% of the window size.

The average estimate of *B. indicus* content for the validated pure *B. indicus* samples increased from .969 (SD = .005) using a window of 25 SNP to .997 (SD = .004) using a window of 500 SNP. The average estimate of *B. taurus* content in validated HF and Jersey animals was close to 1 (.995; SD = .001) when using 25 SNP window and increased slightly to .999 (SD = .001) when using 500 SNP window. The average estimated Jersey content for purebred Jersey animals was .975 (SD = .015) and the HF content for purebred HF animals was .985 (SD = .009), using a 500 SNP window. Increasing window size above 500 SNP did not alter the accuracy of assigning HF *versus* Jersey ancestry. A 500 SNP window was therefore chosen for all analyses. After excluding windows that contained less than 400 SNP, there were 674,972 SNP in 1,346 windows retained.

The ancestral breed origins were then assigned to SNP alleles in crossbred cows based on the inferred origin of the phased haplotypes in which they were located. For two-way (*B. indicus* and *B. taurus*) ancestors, there are four possible combinations of ordered genotypes and four possible combinations of ordered breed origin at each locus. As a consequence, each cow at any given locus will have one of 16 different ancestry genotype combinations (Table 1). Of the 16 combinations, 10 are distinguishable locus states. For three-way (HF, Jersey, and Indigenous) ancestors, there are 36 possible ancestry genotype combinations at each locus (Table 2), of which 24 are distinguishable locus states.

**TABLE 1:** Ancestry genotype combinations for two-way ancestors.

	EE	EI	IE	II
aa	a_E_a_E_	a_E_a_I_	a_I_a_E_	a_I_a_I_
aA	a_E_A_E_	a_E_A_I_	a_I_A_E_	a_I_A_I_
Aa	A_E_a_E_	A_E_a_I_	A_I_a_E_	A_I_a_I_
AA	A_E_A_E_	A_E_A_I_	A_I_A_E_	A_I_A_I_

a and A are the two alleles identified by state at a SNP; I and E indicate that the allele has been tracked back to the indigenous *B. indicus* or the exotic *B. taurus* ancestor respectively.

**TABLE 2:** Ancestry genotype combinations for three-way ancestors.

	HH	HJ	HI	JH	JJ	JI	IH	IJ	II
aa	a_H_a_H_	a_H_a_J_	a_H_a_I_	a_J_a_H_	a_J_a_J_	a_J_a_I_	a_I_a_H_	a_I_a_J_	a_I_a_I_
aA	a_H_A_H_	a_H_A_J_	a_H_A_I_	a_J_A_H_	a_J_A_J_	a_J_A_I_	a_I_A_H_	a_I_A_J_	a_I_A_I_
Aa	A_H_a_H_	A_H_a_J_	A_H_a_I_	A_J_a_H_	A_J_a_J_	A_J_a_I_	A_I_a_H_	A_I_a_J_	A_I_a_I_
AA	A_H_A_H_	A_H_A_J_	A_H_A_I_	A_J_A_H_	A_J_A_J_	A_J_A_I_	A_I_A_H_	A_I_A_J_	A_I_A_I_

a and A are the two alleles identified by state at a SNP; ***I***, ***H*** and ***J*** indicate that the allele has been tracked back to the indigenous, Holstein-Friesian, or Jersey ancestor respectively.

### 2.4 Genome-wide association study

A GWAS for milk yield was undertaken using Wombat software (Meyer, 2007). Firstly, a standard GWAS was undertaken by fitting a single SNP effect regardless of breed origin as follows:

$$y^{*}=1\mu +Xb+{w_{i}g}_{i}+Z_{1}a+Z_{2}h+e,$$ (1)

Where $y^{*}$ is the average adjusted TD milk yield of a cow, μ is the overall mean, X is a design matrix of fixed BC*ENV effects, b is a vector of BC*ENV effects, where BC*ENV is the interaction between breed composition (BC) and production environment (ENV) as defined in (Al Kalaldeh et al., 2021), $w_{i}$ is a vector of genotypes for ${SNP}_{i}$ (coded as 0, 1, or 2), $Z_{1}$ is a design matrix of random additive genetic effects, $Z_{2}$ is a design matrix of random herd effects, $g_{i}$ is the effect size of the i th SNP, a is a vector of random additive genetic effects assumed to be distributed as $∼\;N\left(0,G\sigma_{a}^{2}\right)$ , where G is the genetic relationship matrix constructed using the first method of VanRaden (2008), h is a vector of random herd effects, and e is a vector of residuals.

Secondly, a GWAS was performed to separate the SNP effects into: 1) the effect of *B. indicus versus B. taurus* origin at the locus; 2) the effect of QTL in LD with SNP alleles segregating within the *B. indicus* ancestral breeds; 3) The effect of QTL in LD with SNP alleles segregating within the *B. taurus* ancestral breeds. To do this, a simultaneous regression was performed on the allele count for 1) breed origin (*B. indicus versus B. taurus*), 2) SNP alleles of *B. indicus* origin, and 3) SNP alleles of *B. taurus* origin. The GWAS performed on categories 2) and 3) are equivalent to a within-breed GWAS for *B. indicus* and *B. taurus* respectively, except that they are based on expression in the crossbred cows of QTL that were originally segregating within *B. indicus* and *B. taurus*, respectively.

For the two-ancestor model (*B. indicus* and *B. taurus*), the allele count for breed origin was the number of copies (0, 1 or 2) coming from *B. indicus* ancestor. For SNP alleles of *B. indicus* origin and of *B. taurus* origin that had a MAF > 1%, the following GWAS model was fitted

$$y^{*}=1\mu +Xb+{w1}_{i}\;{g1}_{i}+{w2}_{i}\;{g2}_{i}+{w3}_{i}\;{g3}_{i}+Z_{1}a+Z_{2}h+e,$$ (2)

where ${w1}_{i}$ is a vector of allele count of breed origin for ${SNP}_{i}$ , ${w2}_{i}$ is a vector of genotypes for SNP alleles of *B. indicus* origin for ${SNP}_{i}$ , ${w3}_{i}$ is a vector of genotypes for SNP alleles of *B. taurus* origin for ${SNP}_{i}$ , ${g1}_{i}$ is the effect size of the i th SNP for breed origin, ${g2}_{i}$ is the effect size of the i th SNP for SNP alleles of *B. indicus* origin, ${g3}_{i}$ is the effect size of the i th SNP for SNP alleles of *B. taurus* origin, and the other terms are as defined for Model 1.

For SNP alleles of *B. indicus* origin that had a MAF <1%, only ${g1}_{i}$ and ${g3}_{i}$ were estimated (Model 2a). For SNP alleles of *B. taurus* origin that had a MAF <1%, only ${g1}_{i}$ and ${g2}_{i}$ were estimated (Model 2b).

For three -ancestor (indigenous *B. indicus*, HF, and Jersey) model, GWAS was performed on the allele count for indigenous breed origin, HF breed origin, Jersey breed origin, SNP alleles coming from indigenous ancestor, SNP alleles coming from HF ancestor, and SNP alleles coming from Jersey ancestor. Fitting indigenous breed origin conditional on Jersey breed origin estimates the effects that differentiate indigenous from HF and differentiate Jersey from HF, respectively. Similarly, fitting indigenous breed origin conditional on HF breed origin estimates the effects that differentiate indigenous from Jersey and differentiate HF from Jersey, respectively.

For SNP alleles of indigenous, of HF, and of Jersey origin that had a MAF > 1%, the following GWAS models were fitted:

$$y^{*}=1\mu +Xb+{w1}_{i}\;{g1}_{i}+{w2}_{i}\;{g2}_{i}+{w4}_{i}\;{g4}_{i}+{w5}_{i}\;{g5}_{i}+{w6}_{i}\;{g6}_{i}+Z_{1}a+Z_{2}h+e,$$ (3)

$$y^{*}=1\mu +Xb+{w1}_{i}\;{g1}_{i}+{w2}_{i}\;{g2}_{i}+{w4}_{i}\;{g4}_{i}+{w5}_{i}\;{g5}_{i}+{w7}_{i}\;{g7}_{i}+Z_{1}a+Z_{2}h+e,$$ (4)

Where ${w4}_{i}$ is a vector of genotypes for SNP alleles of HF origin for ${SNP}_{i}$ , ${w5}_{i}$ is a vector of genotypes for SNP alleles of Jersey origin for ${SNP}_{i}$ , ${w6}_{i}$ is a vector of allele count for Jersey breed origin for ${SNP}_{i}$ , ${w7}_{i}$ is a vector of allele count for HF breed origin for ${SNP}_{i}$ , ${g4}_{i}$ is the effect size of the i th SNP for SNP alleles of HF origin, ${g5}_{i}$ is the effect size of the i th SNP for SNP alleles of Jersey origin, ${g6}_{i}$ is the effect size of the i th SNP for Jersey breed origin, ${g7}_{i}$ is the effect size of the i th SNP for HF breed origin, and the other terms are as defined for models 1 and 2.

For SNP alleles of indigenous origin that had a MAF < 1%, only g1 , g4 , g5 , and ( g6 or g7 ) were estimated (Models 3a and 4a). For SNP alleles of HF and Jersey origin that had a MAF < 1%, only g1 , g2 , and ( g6 or g7 ) were estimated (Models 3b and 4b).

After performing GWAS, the genomic inflation factor (*λ*) was calculated for each SNP effect ( g1 to g7 ) as the observed median of chi-squared statistics divided by the expected median of the chi-squared distribution. Due to the degree of correlation between the effects, which can affect the coefficients and *p*-values, the *p*-values were adjusted for genomic control (GC) by dividing the chi-squared test statistics by *λ* so that the median matches the expected value of one. For each effect, the false discovery rate (FDR) was then applied to control the rates of false positive associations.

## 3 Results

### 3.1 The accuracy of breed origin assignment

The accuracy of assigning breed origin to haplotypes in the validated samples of pure *B. indicus* and pure *B. taurus* (HF and Jersey) was very high for all the windows across the genome. The estimated *B. indicus* proportion in the *B. indicus* samples ranged from .953 to 1.00, with an average of .995 (SD = .007). The estimated *B. taurus* proportion in the *B. taurus* samples ranged from .938 to 1.00 with an average of .999 (SD = .004). When HF breed origin was assigned to haplotypes in the HF samples, the estimates ranged from .850 to 1.00, with an average of .983 (SD = .025), whereas the Jersey proportion in the Jersey samples ranged from .750 to 1, with an average of .975 (SD = .034).

### 3.2 Allele frequencies of breed origin alleles

For the two-ancestor model, the frequency of the I alleles for *B. indicus* breed origin in crossbred cows ranged from .30 to .48 across windows, with an average of .36 (SD = .01). The allele count for I alleles from the two-ancestor model was very similar to those obtained from the three–ancestor model, with an average correlation of .995 (ranging from .92 to 1.00) across all windows. The frequency of HF alleles ranged from .36 to .56 with an average of .49 (SD = .02) across windows, whereas the frequency of JR alleles ranged from .08 to .27 with an average of .15 (SD = .02).

### 3.3 Allele frequencies of SNP alleles of ancestral origin

**FIGURE 1:**
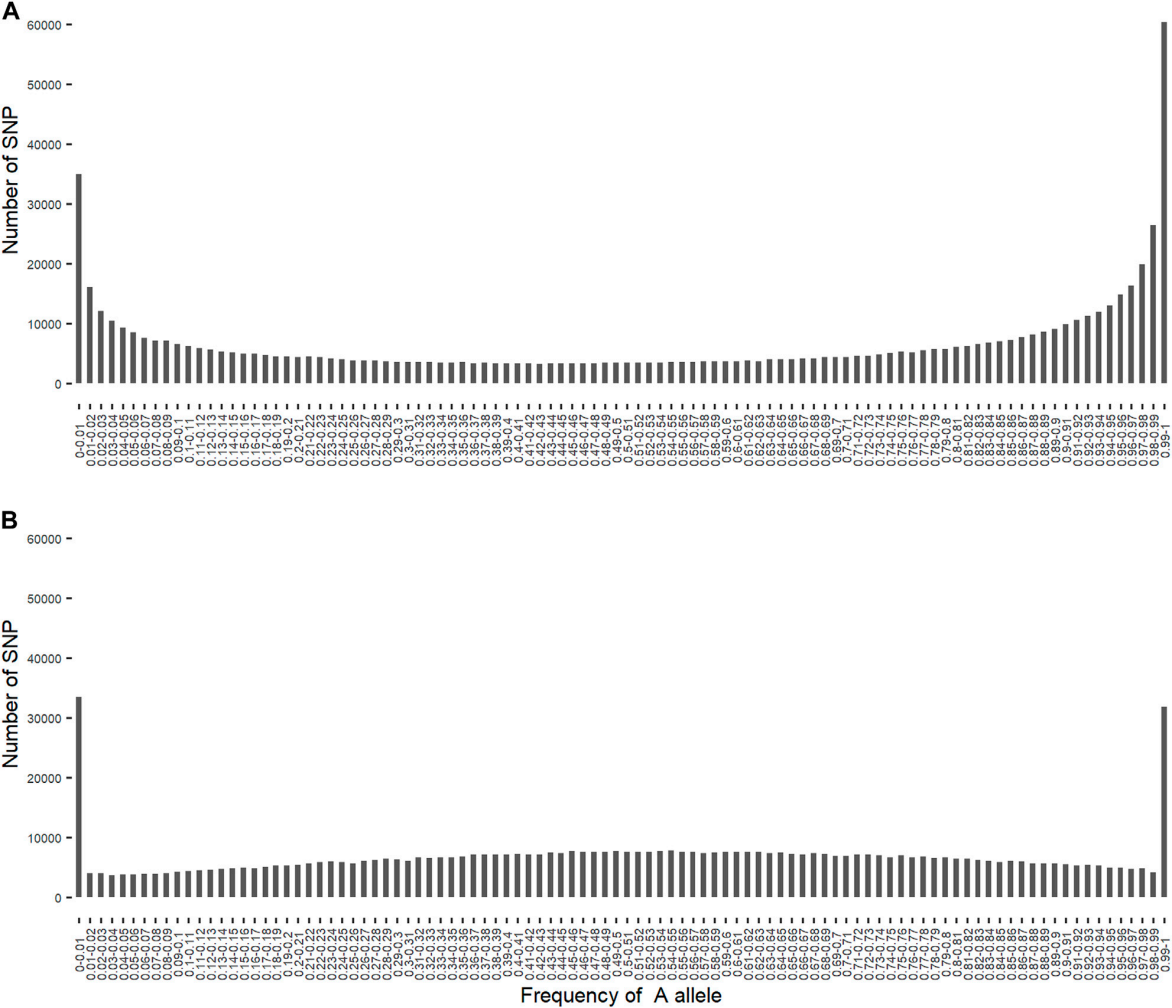
Distribution of the frequency of A allele for SNP alleles of indigenous *B. indicus* origin (Figure 1A) and of exotic *B. taurus* origin (Figure 1B).

As expected, the frequency of the A allele for SNP alleles of indigenous *B. indicus* origin and of exotic *B. taurus* origin in crossbred cows ranged from zero to one (Figure 1). There were 95,229 and 64,996 SNP alleles of indigenous origin and of exotic origin that had a MAF < 1%, respectively. Excluding SNP alleles with a MAF < 1%, the distribution of SNP alleles of indigenous origin remains skewed toward low MAF, whereas the distribution of SNP alleles of exotic origin is skewed toward high MAF. Similar results were also reported for African crossbred cattle by Aliloo et al. (2018) and Strucken et al. (2017), and is attributed to bias in selection of SNP on the genotyping assays.

The frequency of A allele for SNP alleles of indigenous origin, of HF origin, and of Jersey origin using three-way ancestors, also ranged from zero to one (Supplementary Figure S1). There were 68,865 and 101,099 SNP alleles of HF origin and of Jersey origin that had a MAF < 1%, respectively. Of which, 51,606 SNP alleles had a MAF < 1% in both HF and Jersey origin, and the remaining SNP alleles were skewed towards low MAF. There were 97,108 SNP alleles of indigenous origin that had a MAF < 1%, of which 1,993 SNP alleles of HF origin and 14,230 SNP alleles of Jersey origin also had a MAF < 1%, all being of loci whose raw genotypes had low MAF (<.02).

### 3.4 A standard GWAS

**FIGURE 2:**
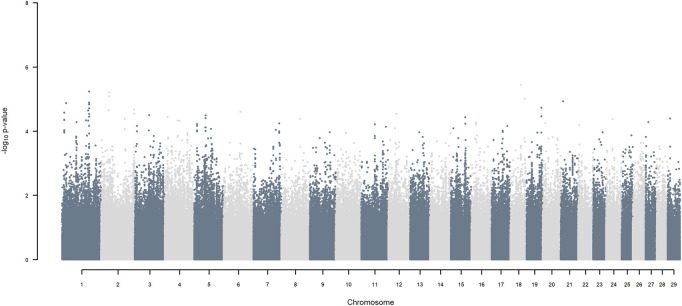
Manhattan plot of GWAS (Model 1) in which each SNP genotype was tested for an association with milk yield.

GWAS, in which each SNP genotype was tested for an association with milk yield, was undertaken. NO SNP exceeding an FDR of 30% was detected (Figure 2).

### 3.5 GWAS using two ancestors

**FIGURE 3:**
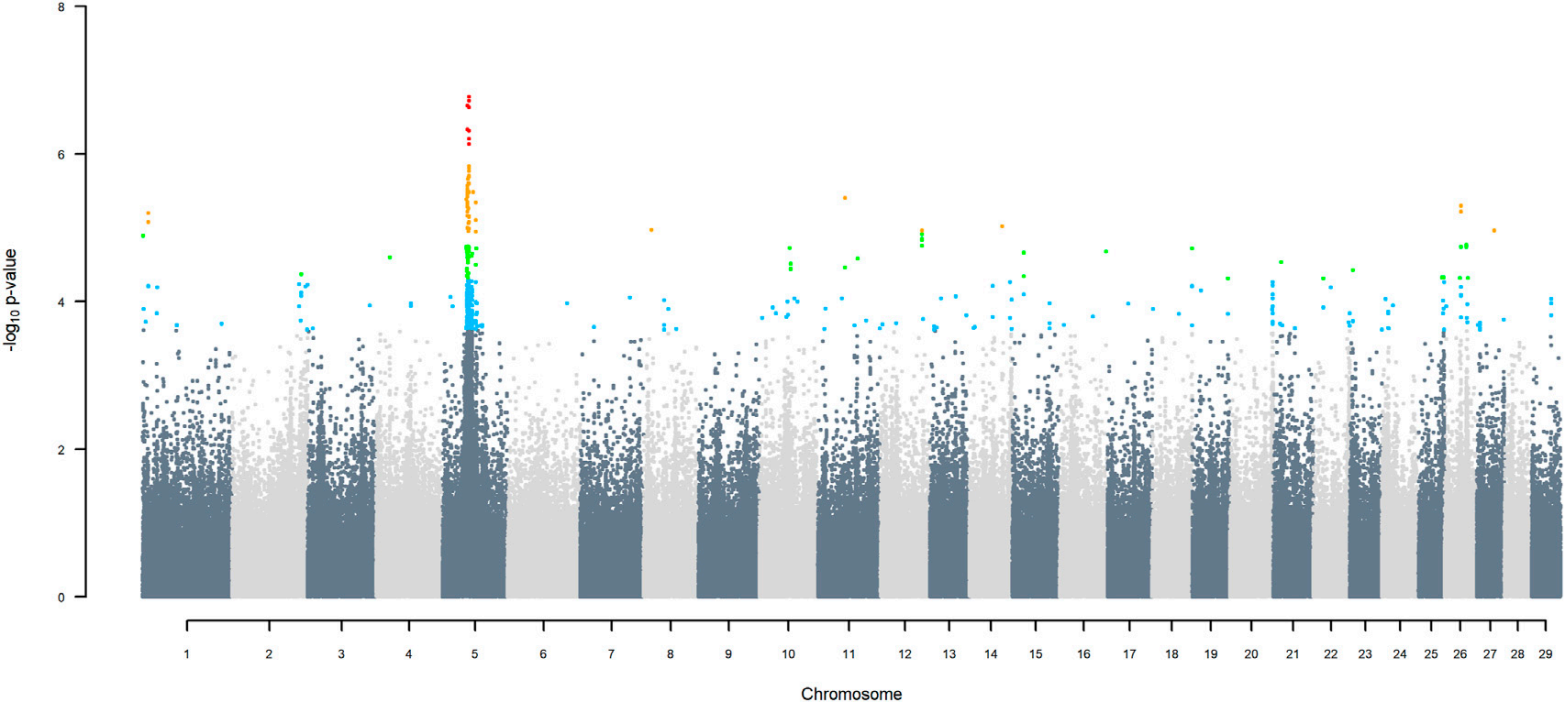
Manhattan plot of GWAS (Model 2) for indigenous *versus* exotic origin using SNP alleles of indigenous and of exotic origin that had a MAF > 1%. SNP that passed the FDR threshold of 5%, 10%, 20%, and 30% are highlighted in red, orange, green, and blue, respectively.

**FIGURE 4:**
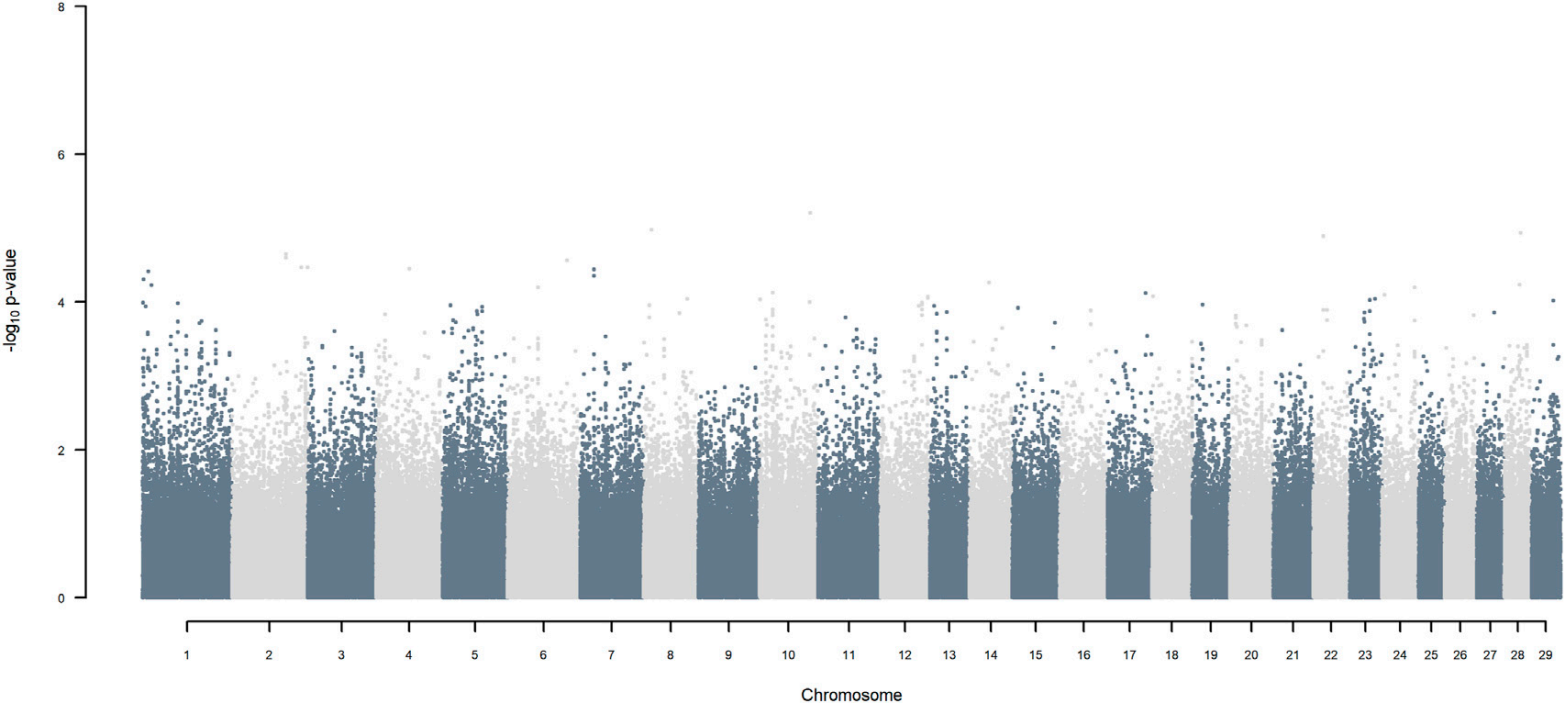
Manhattan plot of GWAS (Model 2) for SNP alleles of indigenous origin using SNP alleles of indigenous and of exotic origin that had a MAF > 1%.

**FIGURE 5:**
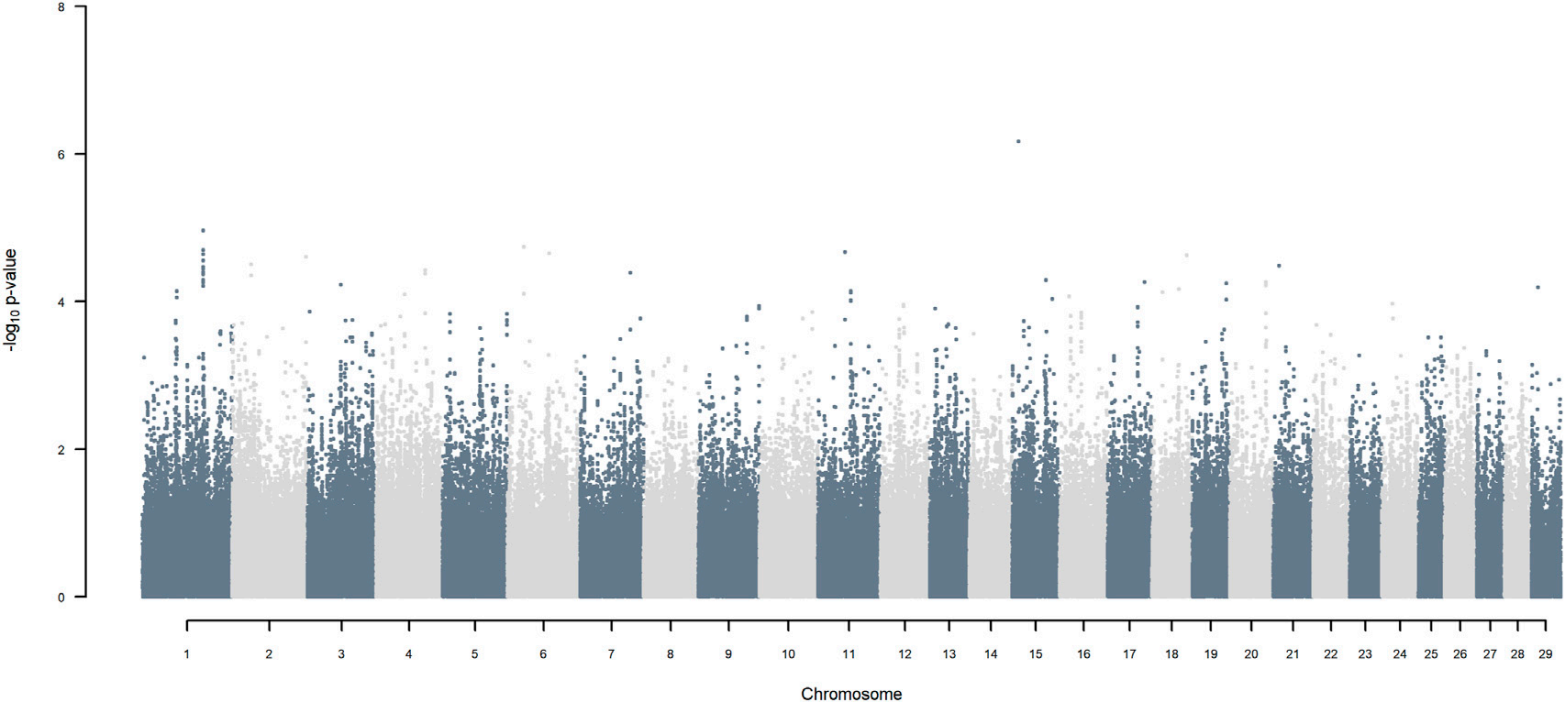
Manhattan plot of GWAS (Model 2) for SNP alleles of exotic origin using SNP alleles of indigenous and of exotic origin that had a MAF > 1%.

GWAS for SNP alleles tracking exotic *versus* indigenous origin identified a region between 44.63 and 47.37 Mbp on BTA5 for breed origin, within which 9 SNP reached an FDR level of 5% (Figure 3), with the indigenous alleles increasing milk yield. The identified region corresponds to three haplotype windows, located between 43.98 and 49.44 Mbp, with 100% accuracy for assigning indigenous *versus* exotic origin. Considering a less conservative FDR of 30%, a substantial proportion of the genome passed the threshold for breed origin effects (Figure 3), with positive effects on milk yield coming from both indigenous and exotic ancestors. There were no SNP significant at FDR 30% for QTL segregating within indigenous or within exotic ancestors (Figures 4,5).

The region for indigenous origin effect on BTA5 was also found to be significant at FDR 5% when GWAS was performed for SNP alleles of exotic origin that had a MAF < 1% (Model 2b) when SNP alleles of indigenous origin had a MAF > 1%. However, the region only reached FDR 20% when GWAS was performed for SNP alleles of indigenous origin that had a MAF < 1% (Model 2a) when SNP alleles of exotic origin had a MAF > 1% (results not shown).

### 3.6 GWAS using three ancestors

Fitting indigenous breed origin conditional on Jersey breed origin (GWAS Model 3) estimates the difference of indigenous *versus* HF origin and the difference of Jersey *versus* HF origin, respectively. Likewise, fitting indigenous breed origin conditional on HF breed origin (GWAS Model 4) estimates the difference of indigenous *versus* Jersey origin and the difference of HF *versus* Jersey origin, respectively. Therefore, Jersey breed origin (GWAS Model 3) and HF breed origin (GWAS Model 4) give identical estimates of SNP effects but with opposite sign. Similarly, the effects of SNP alleles of HF, of Jersey, and of indigenous origin (i.e., the GWAS based on within-ancestor linkage disequilibrium) are identical for Model 3 and Model 4.

**FIGURE 6:**
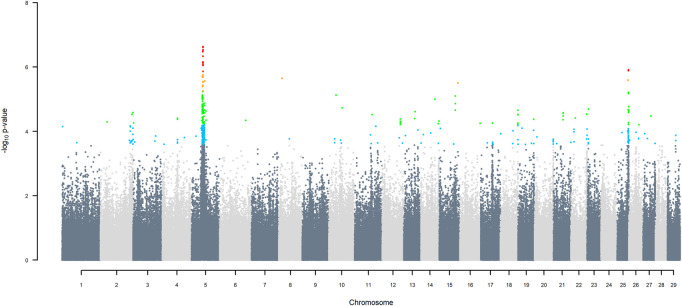
Manhattan plot of GWAS (Model 3) for indigenous *versus* HF origin using SNP alleles of all origins that had a MAF > 1%. SNP that passed the FDR threshold of 5%, 10%, 20%, and 30% are highlighted in red, orange, green, and blue, respectively.

**FIGURE 7:**
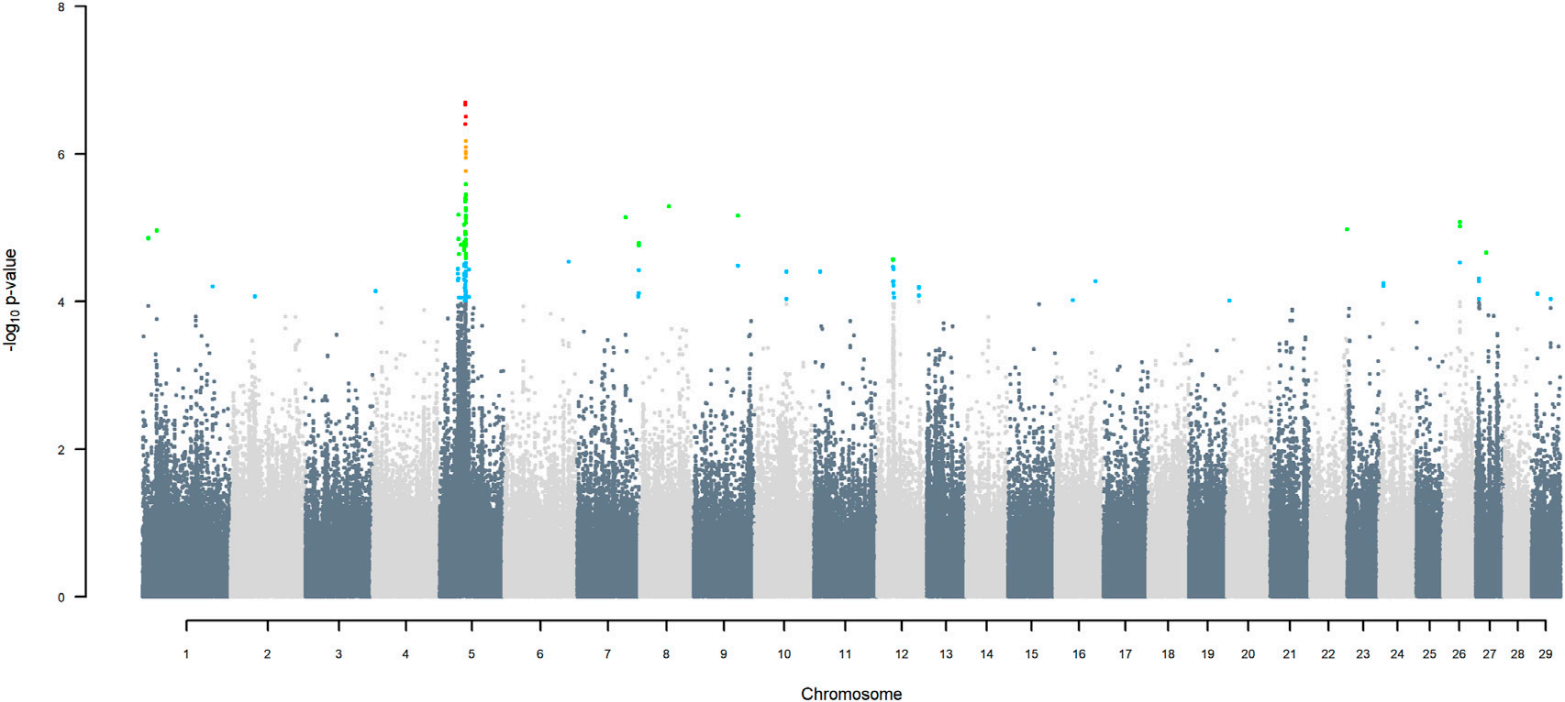
Manhattan plot of GWAS (Model 4) for indigenous *versus* Jersey origin using SNP alleles of all origins that had a MAF > 1%. SNP that passed the FDR threshold of 5%, 10%, 20%, and 30% are highlighted in red, orange, green, and blue, respectively.

**FIGURE 8:**
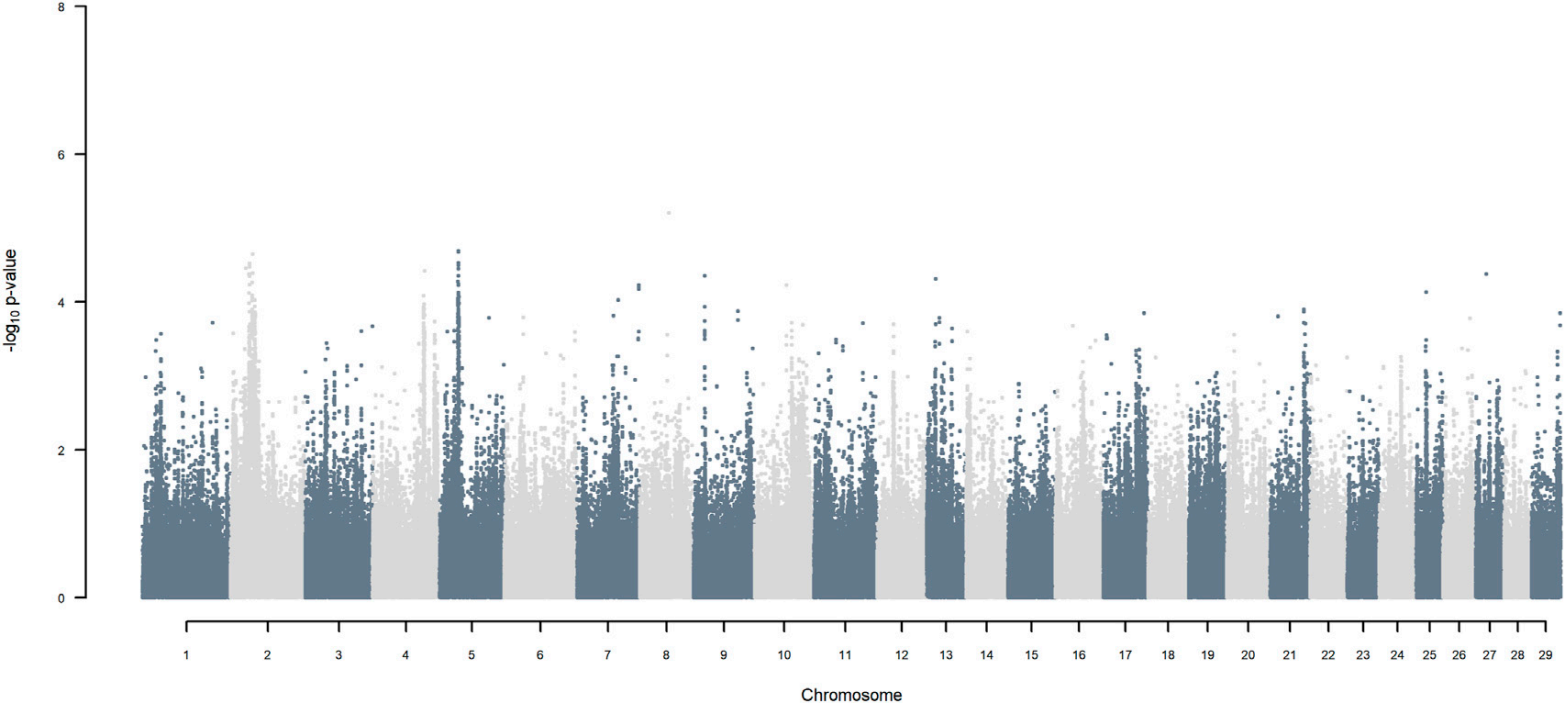
Manhattan plot of GWAS (Models 3 & 4) for HF *versus* Jersey origin using SNP alleles of all origins that had a MAF > 1%.

**FIGURE 9:**
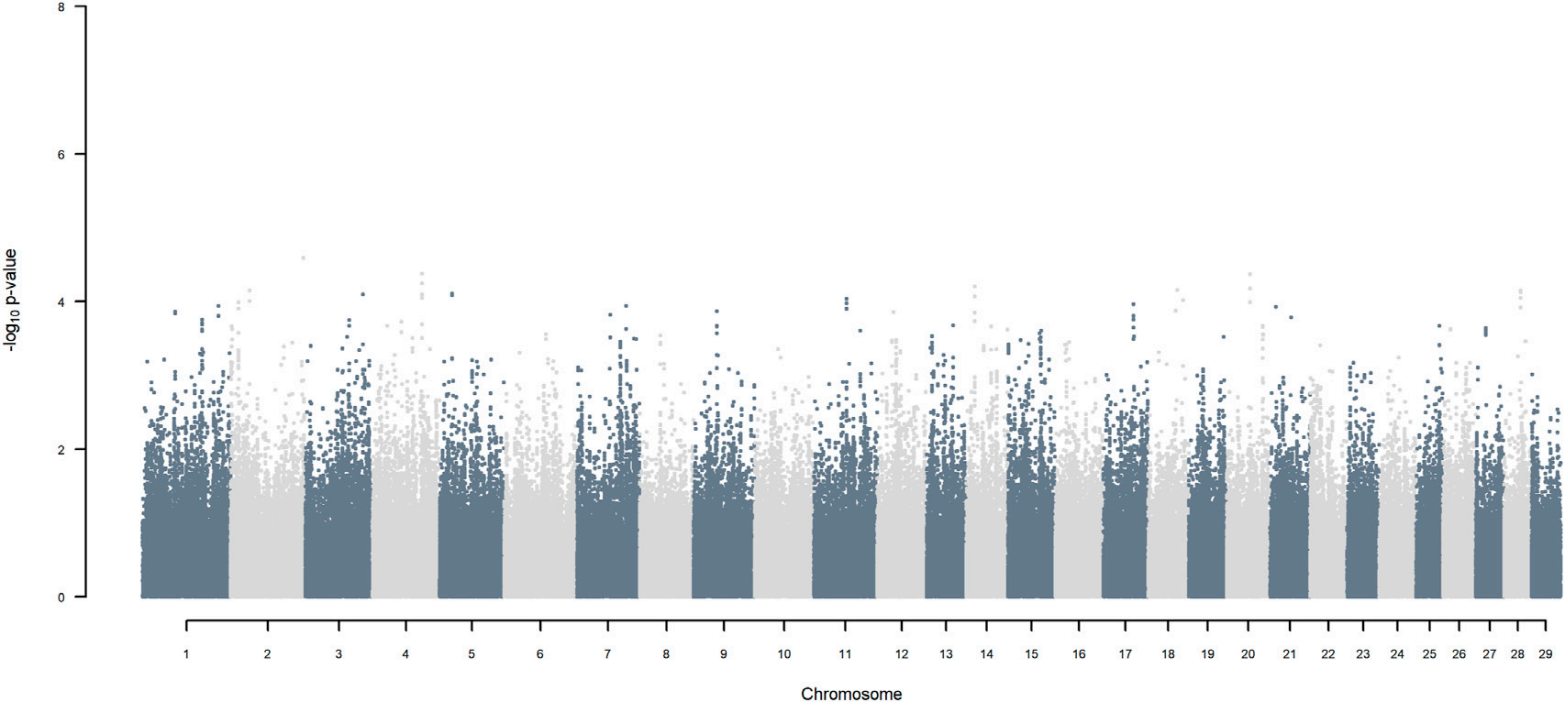
Manhattan plot of GWAS (Models 3 & 4) for SNP allele of HF origin using SNP alleles of all origins that had a MAF > 1%.

**FIGURE 10:**
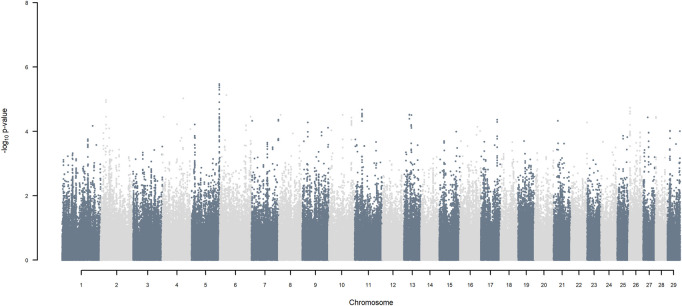
Manhattan plot of GWAS (Models 3 & 4) for SNP allele of Jersey origin using SNP alleles of all origins that had a MAF > 1%.

**FIGURE 11:**
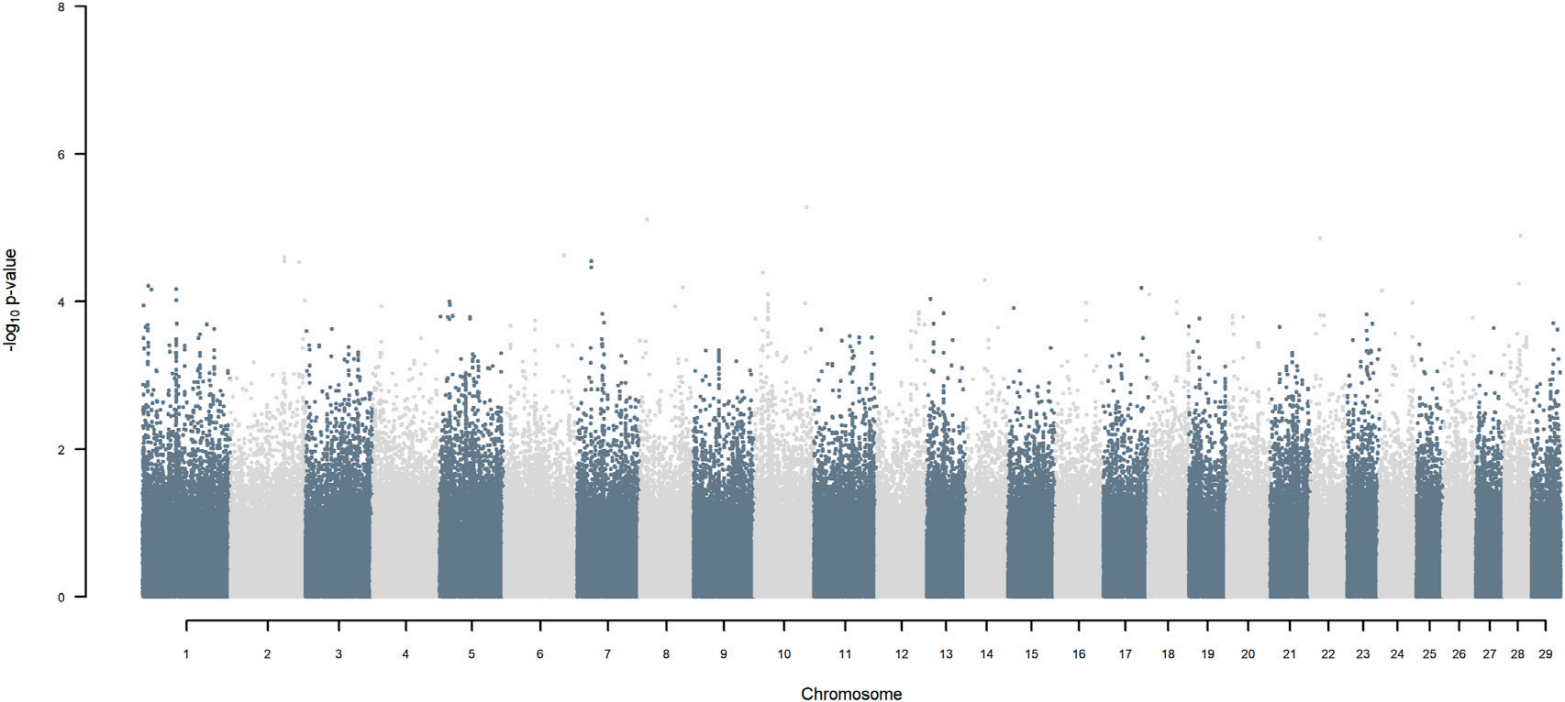
Manhattan plot of GWAS (Models 3 & 4) for SNP allele of indigenous origin using SNP alleles of all origins that had a MAF > 1%.

Manhattan plots of GWAS using three ancestors for SNP alleles of all origins that had a MAF > 1%, are given in (Figures 6–11) The region for indigenous *versus* exotic breed origin detected on BTA5 using the two-ancestor model, was also found to be significant at FDR 5% using the three-ancestor model, differentiating indigenous from HF (GWAS Model 3; Figure 6) and differentiating indigenous from Jersey (GWAS Model 4; Figure 7). The same region was also found to be significant at FDR 5% when GWAS Models 3b & 4b were performed for SNP alleles of indigenous origin that had a MAF > 1% when SNP allele of HF and Jersey origin had a MAF < 1% (Supplementary Figures S2, S3). In the validation test, the accuracy of assigning indigenous, HF and Jersey origin to alleles in this region on BTA five using the three-ancestor model was 100%.

GWAS Model three using SNP alleles of all origins that had a MAF > 1% also detected two significant SNP with indigenous *versus* HF origin effect, at 39.76 and 40.25 Mbp on BTA25, at FDR of 5% (Figure 6), with the positive effect coming from HF. GWAS Model 3a performed on SNP allele of HF and of Jersey origin that had a MAF > 1%, when SNP alleles of indigenous origin had a MAF < 1%, detected a significant SNP (FDR < 5%) in the same region at 40.79 Mbp (Supplementary Figure S4). These significant SNP correspond to two haplotype windows located between 38.85 and 42.19 Mbp, that had 100% accuracy of assignment of indigenous and Jersey alleles and 95% accuracy for HF alleles.

Considering a less conservative FDR of 30%, a large number of indigenous *versus* HF and indigenous *versus* Jersey breed origin effects passed the threshold (Figures 6,7), with alleles of positive effect on milk yield coming from all three ancestral breeds. No estimates for the differences between HF and Jersey origin exceeded an FDR of 30% (Figure 8). No estimates for SNP alleles within indigenous, within HF and within Jersey exceeded an FDR of 30% (Figures 9–11).

## 4 Discussion

A standard genome wide association analysis (GWAA) detects linkage disequilibrium (LD) between SNP alleles and a QTL within a population. In a crossbred population resulting from n ancestors, there are 2n−1 possible forms of LD: the LD that existed within each of the n ancestors that is transmitted directly to their crossbred descendants, plus n−1 forms of LD with the newly created QTL, which are the loci that cause the functional differences between the ancestors, that were fixed or nearly fixed for a single QTL allele in the ancestral populations. Thus, while a single GWAA can be performed in a crossbred population, its interpretation is obscure. Moreover, it can have substantially lower power to detect QTL that were segregating in the ancestral populations, than undertaking a GWAA within pure ancestor populations, because the direction and size of LD is expected to vary between all but very closely related ancestral populations. The result is that no locus is expected to exhibit LD as high as in some of the ancestor populations. Several studies utilized breed origin information in their GWAS models (Bolormaa et al., 2013; Otto et al., 2019; Aliloo et al., 2020), however, without fully accounting for all possible forms of LD (within and between ancestral populations) simultaneously in crossbred populations. To the best of our knowledge, this study is the first that aimed to account for loci that functionally differentiate the ancestral populations in addition to detecting QTL segregating within each of the ancestral populations in crossbred cows.

If, as done here, haplotypes of crossbred individuals can be traced back to their ancestral origin, it is possible to undertake a GWAA that simultaneously accounts for loci that functionally differentiated the ancestral populations in addition to detecting QTL that were segregating within each of the ancestral populations. Considering the simplest situation of a crossbred population resulting from equal contributions of two ancestral populations, and assuming, as observed here, that haplotypes are assigned to ancestral origin with close to 100% accuracy, the power for detecting the presence of loci that functionally differentiate the two ancestors is maximal. This is because the allele frequency of ancestral origin is .5 and the LD with linked SNP is close to one over large genomic distances because of low frequency of recombination events. The power of detecting QTL segregating within the ancestral populations is substantially lower in the crossbred population than in a similarly sized purebred population because only half the alleles in the crossbred population come from one of the two ancestors. In our crossbred data, the admixture analyses estimated the ancestral origin to be approximately .36 *B. indicus* and .64 *B. taurus*, made up of approximately .49 HF and .15 Jersey. As the power for detecting a QTL is proportional to m (1−m) where m is the minor allele frequency of the SNP (e.g., Visscher et al., 2017), the power to detect functional loci fixed for opposite alleles in *B. indicus* vs. *B. taurus,* is 92% of maximum possible power (when m = .5). The power to detect additive QTL segregating within indigenous, within HF and within Jersey ancestral populations, is equivalent to undertaking GWAA in each of the pure populations of approximately .36, .49 and .15 the size of the crossbred population analysed here. Thus, for example, the power of detecting QTL segregating within *B. indicus* is equivalent to undertaking a GWAA on about 1,800 purebred *B. indicus* cattle, compared to the approximately 5,000 crossbred cattle analysed here. Furthermore, the power to detect QTL segregating within indigenous ancestors is lower than within HF and Jersey due to the lower average MAF of SNP within *B. indicus* (Figure 2).

The indigenous cattle that are used to create the current crossbred dairy population are not a single population across the regions sampled in this study. Farmers typically use Desi (non-descript) indigenous cattle when producing crossbred animals rather than pure indigenous breeds. There has been little study of the variation of Desi cattle across India but visually they differ greatly between and even within the regions included in this study, and certainly cannot be considered as a single homogeneous breed. As such it is likely that LD between SNP and QTL will vary substantially between different populations of Desi, further reducing the power to detect such QTL in this crossbred population. We were unable to differentiate different sources of Desi using SNP marker data because of the remarkably low SNP variation observed between *B. indicus* breeds. Approximately 1% of all SNP variation within *B. indicus* occurs between breeds*,* compared to the nearly 40% of variation within *B. taurus* being between *B. taurus* breeds (Strucken et al., 2021). The GWAA for QTL segregating within *B. taurus* in the two-ancestor model, or within HF and Jersey in the three-ancestor model, had a power equivalent to undertaking a GWAA in approximately 3,200, 2,450 and 750 purebred cows, respectively. Although these numbers seem quite substantial for *B. taurus* and HF, no QTL regions were detected at FDR 30%. A contributing factor will have been the relatively lower heritability and genetic variation of milk yield in this population (h^2^ ∼ .18) compared to typical intensive dairy systems in which most GWAA have been undertaken. This lower variation reflects the difficulty of obtaining frequent and accurate data in smallholder dairy systems, plus the substantial environmental fluctuations over time in smallholder systems, which are also more difficult to account for statistically because of the low herd size (Al Kalaldeh et al., 2021).

Although the power is high to detect loci differentiating *B. indicus* from *B. taurus* ancestral breeds, the estimate of location has low accuracy compared to a GWAA within a typical purebred population. This is because in recently created crossbred populations there have been few recombination events and LD between the newly created QTL and the ancestral origin of SNP remains very high over large genomic regions. The situation is essentially the same as the early days of QTL mapping in segregating populations, using techniques such as Haley-Knott regression (Haley and Knott, 1992). Large scale use of crossbred dairy cattle in India began about 50 years ago; approximately eight cattle generations. But crossbreeding in India typically occurs by mating existing crossbred cows owned by smallholders to either purebred or F1 or first-generation backcross bulls. So, haplotypes will range from those that result from many generations of recombination to a majority with rather few, including zero, generations of recombination. Thus, LD is expected to remain high over long distances and the confidence intervals of the location of loci differentiating *B. indicus versus B. taurus* are expected to remain large.

This is reflected in the large region between 43.98 and 49.44 Mbp on BTA5 that differentiates *B. indicus* from *B. Taurus*, even when restricting the putative interval to the range of those SNP with FDR > 5%. The true confidence interval is undoubtedly larger. Assigning breed origin to haplotypes in this region was very accurate (100%), not just for *B. taurus* and *B. indicus* alleles but also for alleles of HF and Jersey origin. The lack of selection at this region within the crossbred population is confirmed by the frequency of *B. indicus* origin alleles ranging from .348 to .365, which is very similar to the average of *B. indicus* origin allele across the whole genome. GWAA using three ancestors confirmed that this region on BTA5 differentiates indigenous from HF and indigenous from Jersey, with the indigenous alleles increasing milk yield. The estimated effect is expected to have positive ascertainment bias. Nevertheless, the average difference between homozygous indigenous *versus* exotic in this region is .85 kg/day (SD = .05), which is about 11% of the average TD milk yield. If a large effect is confirmed in subsequent validation studies, this and other loci differentiating *B. indicus* from *B. taurus* effects on milk yield may prove valuable for genetic selection of crossbreds.

GWAA using a three-ancestor model detected an indigenous *versus* HF origin effect between 39.76 and 40.25 Mbp on BTA25 at FDR 5%, with the HF alleles increasing milk yield. The average difference between homozygous indigenous and HF in this region is .52 kg/day (SD = .04), being ∼7% of the average yield. In the same region, no effect was detected between indigenous and Jersey nor between HF and Jersey. So, at this stage it cannot be determined whether this region might indicate a milk-increasing genetic variant that is unique to HF, or whether it is, like the region on BTA5, a *B. indicus versus B. taurus* difference.

A striking result from the current analyses is the large number of regions that differentiate indigenous from exotic that were detected at FDR 30%. Also notable is that variants that increase milk yield in crossbreds come from both indigenous and exotic ancestors. While recognizing that detection of such loci has substantially higher power than detection of QTL segregating within the ancestral populations, the large number of loci detected compared to no QTL detected segregating within ancestral populations, suggests that breed origin loci may, on average, be of larger effect than within-breed QTL. Furthermore, no regions differentiating HF from Jersey reached the FDR30% compared to a large number of regions differentiating indigenous from exotic that reached the FDR30% due to the relatively lower power to detect loci differentiating HF from Jersey compared to between indigenous and exotic breeds. This is due to much lower genetic variation within *B. taurus* breeds compared to between *B. indicus* and *B. taurus* breeds (Strucken et al., 2021).

Confirmation awaits more data from these Indian crossbred populations, which will be collected in the next several years, or analysis of other populations. The Girolando, which is a hybrid of Holstein and Gir, used widely in Brazil and for which high quality data recording exists, would be an interesting population to analyse (Canaza-Cayo et al., 2018). A difference with the current study is that the Gir is a well-recognised *B. indicus* dairy breed, while the *B. indicus* ancestors of crossbreds in our study are mostly Desi (non-descript) cattle rather than indigenous dairy breeds. If some of the same genomic regions have been selected to produce both *B. indicus* and *B. taurus* dairy breeds, they will not show up in an analysis of Girolando data.

Because of the large confidence intervals attached to all putative breed origin effects identified in this study, we only discuss the largest effects detected, on BTA5 and BTA25. The region identified on BTA5 was previously reported as having been under selection in several Indian *B. indicus* breeds (Dixit et al., 2020) and in African zebu and *B. indicus* (Gir) cattle (Tijjani et al., 2021). Naval-Sánchez et al. (2020) and Sun et al. (2022) recently compared the genome sequence of *B. taurus* and *B. indicus* cattle and reported missense mutations in *HELB* gene, located around 47.73 Mb on BTA5, that were specific to *B. indicus* cattle, which they suggested was an adaptation to hot environments, given that *HELB* is involved in DNA damage response. The BTA5 region also harbours *DYRK2* and *RAP1B* genes with functions related to mammary gland development and lactation. *DYRK2* is a member of a family of protein kinases that regulate the lactating mammary gland differentiation and development (Edwards et al., 1998; Chodosh et al., 2000) and has been reported as a candidate gene for udder support scores, teat length, and teat diameter in B indicus*B taurus crossbred beef cows (Tolleson et al., 2017). *RAP1B* is a highly conserved milk gene across Mammalia (Lemay et al., 2009). The high expression of *RAP1B* in the bovine mammary gland was found to be associated with mastitis resistance (Lawless et al., 2014). However, *B. indicus* ancestry in our crossbred populations is expected to be from Desi (non-descript) cattle rather than from indigenous dairy breeds. Given that it is the *B. indicus* allele that increases milk yield in this region, it may be more likely that the causal gene is involved in environmental adaptation that allows expression of the milk production potential of crossbreds, rather than a gene that directly causes increased milk production.

The region identified on BTA25, with the increasing allele coming from the *B. taurus* ancestor, is contained within a QTL region previously reported for persistency of milk yield in Holstein cattle (Harder et al., 2006), and overlaps with a region for milking speed in French Holstein cattle (Marete et al., 2018) and North American Holstein cattle (Chen et al., 2020). Previous GWAS had also reported this region to be associated with fertility and reproduction traits in Holstein cattle (e.g., Jaton et al., 2018; Chen et al., 2022).

## 5 Conclusion

A large number of genomic regions that define ancestral differences for milk yield between *B. indicus* and *B. taurus* were detected across the genome, with variants that increase milk yield coming from both indigenous and exotic ancestors, and with some effects being of large size. Although acknowledging the weaker power for detecting genetic variation within the ancestral breeds, and the need for validation, these results suggest that genetic variation between ancestral breeds that now segregates in crossbred dairy cattle, may be important for future genomic selection of smallholder crossbred dairy cattle.

## Data Availability

The data analyzed in this study is subject to the following licenses/restrictions: The data generated specifically for this study were collected within the Enhanced Genetic Gains program (EGP) of BAIF Development Research Foundation (baif.org.in) and are available upon reasonable request. Requests to access these datasets should be directed to MS, mswami@baif.org.in.

## Supplementary material

SUPPLEMENTARY FIGURE S1 Distribution of allele frequencies of SNP of indigenous origin **(A)**, of HF origin **(B)** and of Jersey origin **(C)** using three-way ancestral assignment.

SUPPLEMENTARY FIGURE S2 Manhattan plot of GWAS (Model 3b) for indigenous versus HF origin using SNP alleles of indigenous origin that had a MAF > 1% when SNP allele of HF and Jersey origin had a MAF < 1%. SNP that passed the FDR threshold of 5%, 10%, 20%, and 30% are highlighted in red, orange, green, and blue, respectively.

SUPPLEMENTARY FIGURE S3 Manhattan plot of GWAS (Model 4b) for indigenous versus Jersey origin using SNP alleles of indigenous origin that had a MAF > 1% when SNP allele of HF and Jersey origin had a MAF < 1%. SNP that passed the FDR threshold of 5%, 10%, 20%, and 30% are highlighted in red, orange, green, and blue, respectively.

SUPPLEMENTARY FIGURE S4 Manhattan plot of GWAS (Model 3a) for indigenous versus HF origin using SNP allele of HF and of Jersey origin that had a MAF > 1% when SNP alleles of indigenous origin had a MAF < 1%. SNP that passed the FDR threshold of 5%, 10%, 20%, and 30% are highlighted in red, orange, green, and blue, respectively.
