# Supplementary figures and images for: Detection of genomic regions that differentiate Bos indicus from Bos taurus ancestral breeds for milk yield in Indian crossbred cows
Source: Front Genet. 2023 Jan 9;13:1082802. doi: 10.3389/fgene.2022.1082802 (PMC9868639; doi:10.3389/fgene.2022.1082802)

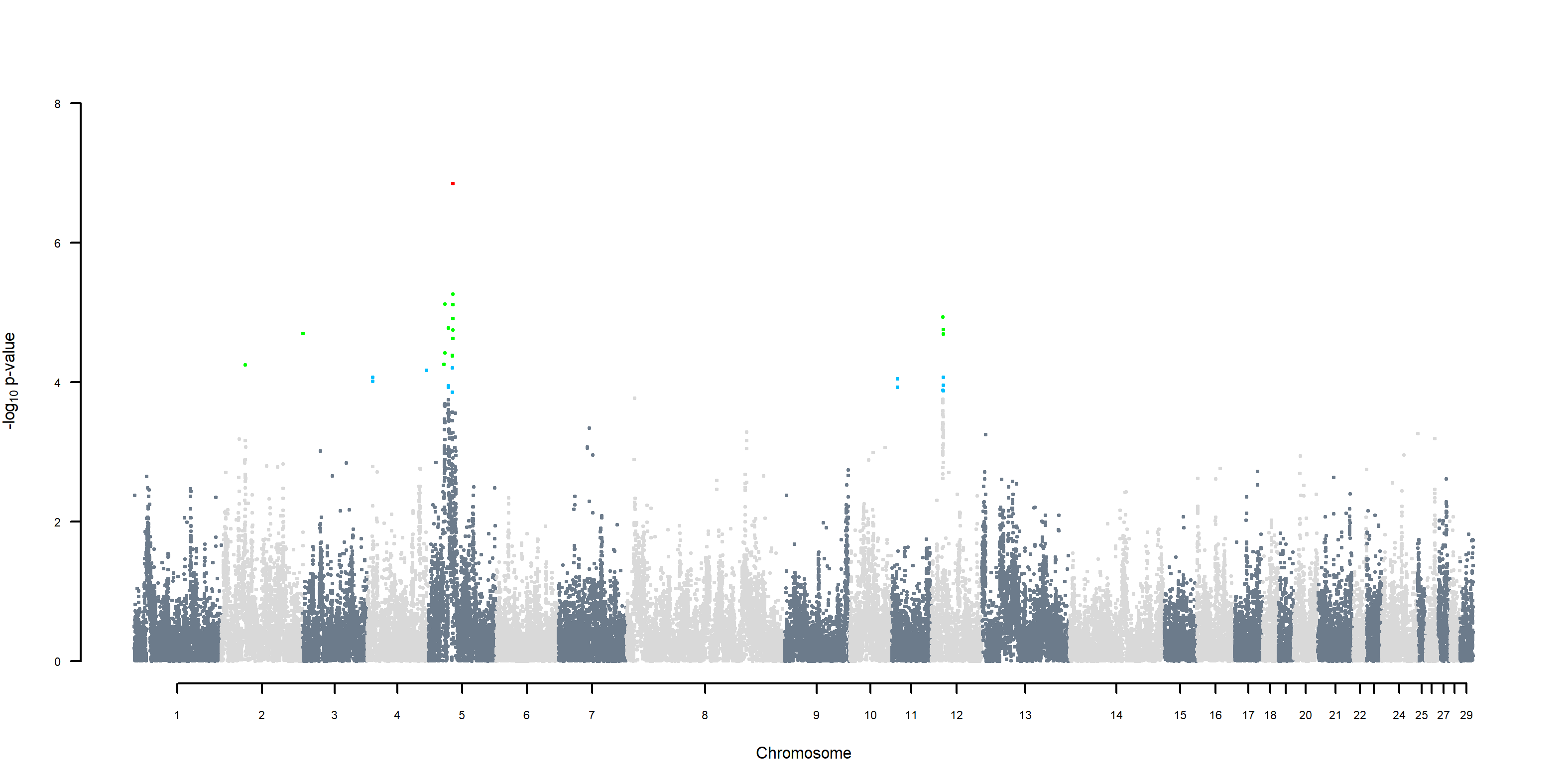

Supplement: Supplementary file 1 [file Image3.TIFF]

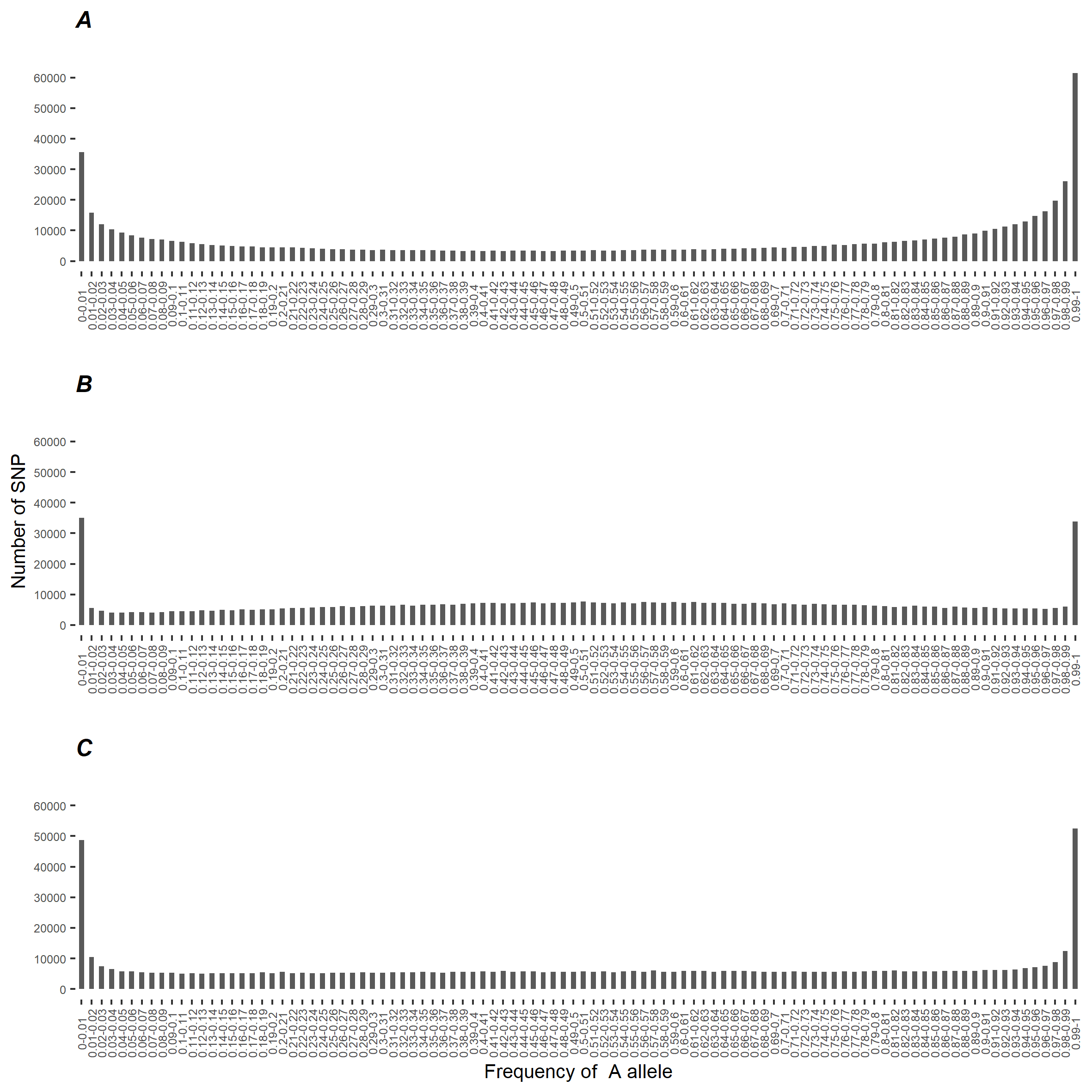

Supplement: Supplementary file 2 [file Image1.TIFF]

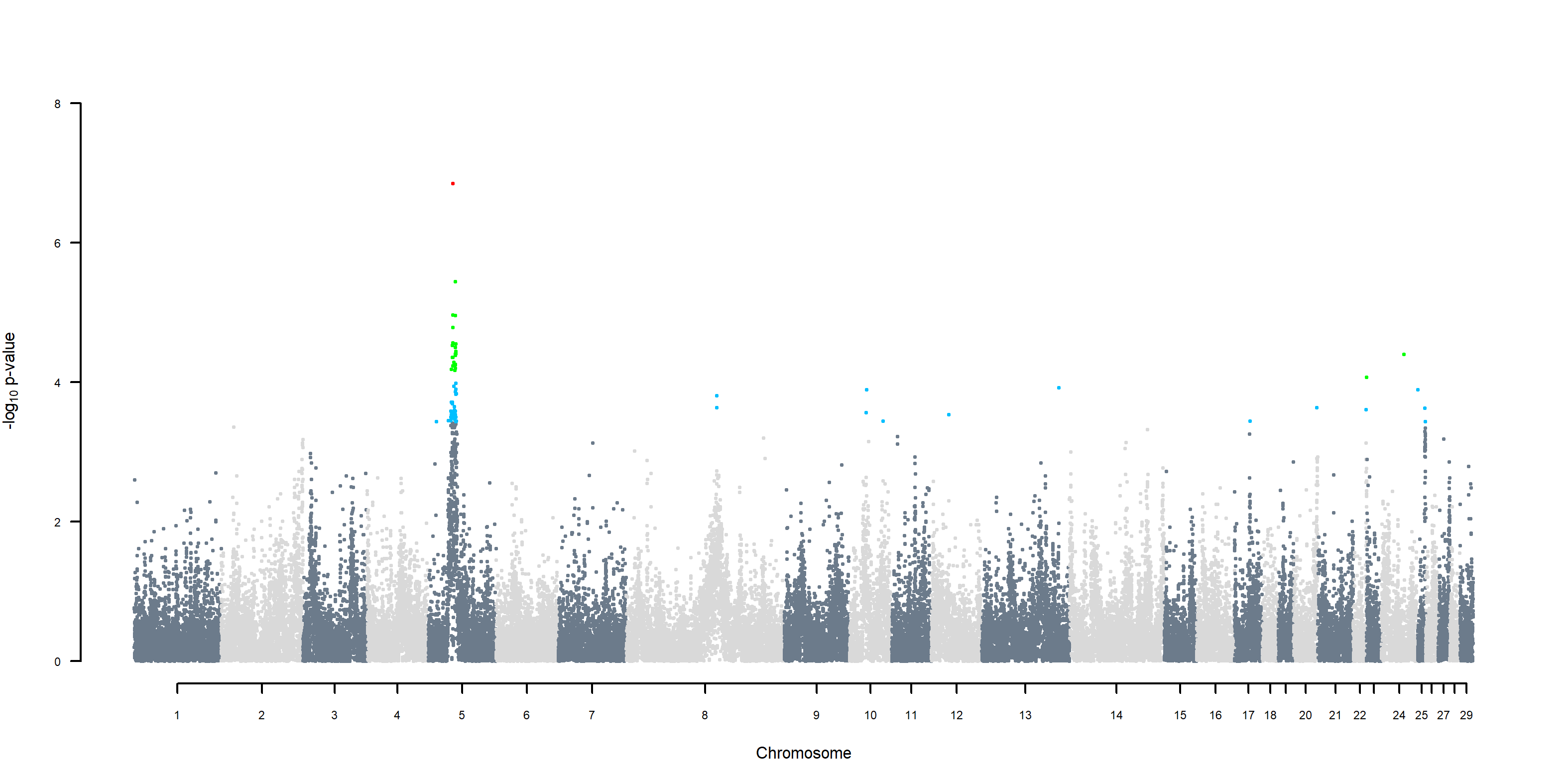

Supplement: Supplementary file 3 [file Image2.TIFF]

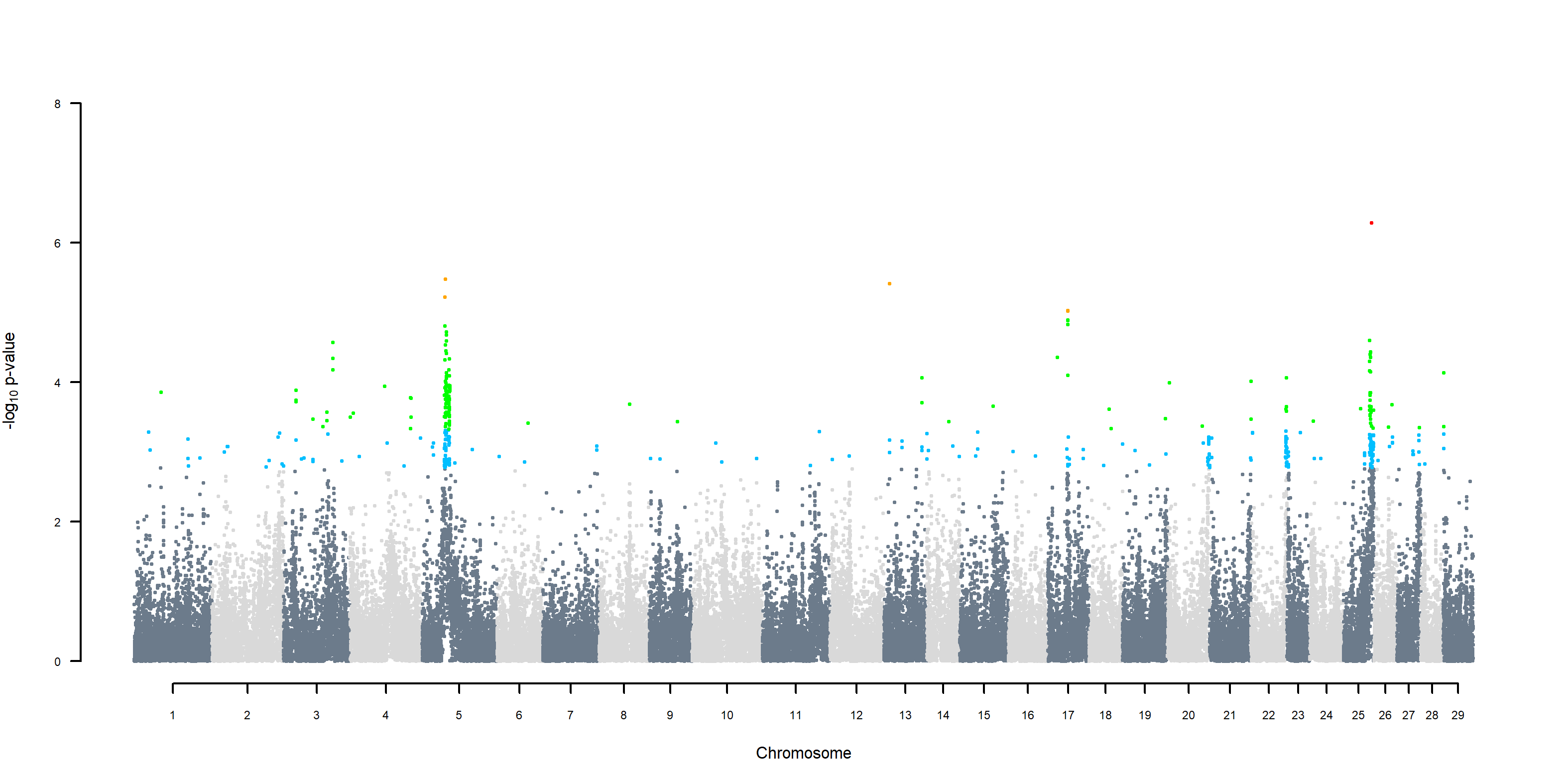

Supplement: Supplementary file 4 [file Image4.TIFF]
